# Microglia-mediated inflammatory destruction of neuro-cardiovascular dysfunction after stroke

**DOI:** 10.3389/fncel.2023.1117218

**Published:** 2023-03-21

**Authors:** Jiahong Deng, Chenghan Chen, Shuaishuai Xue, Daoqing Su, Wai Sang Poon, Honghao Hou, Jun Wang

**Affiliations:** ^1^Department of Neurosurgery, Nanfang Hospital, Southern Medical University, Guangzhou, China; ^2^The First School of Clinical Medicine, Southern Medical University, Guangzhou, China; ^3^Neural Networks Surgery Team, Southern Medical University, Guangzhou, China; ^4^Department of Neurosurgery, The First Affiliated Hospital of Fujian Medical University, Fuzhou, Fujian, China; ^5^Neuro-Medicine Center, The University of Hong Kong-Shenzhen Hospital, Shenzhen, China; ^6^Guangdong Provincial Key Laboratory of Construction and Detection in Tissue Engineering, School of Basic Medical Science, Southern Medical University, Guangzhou, China

**Keywords:** microglia, stroke, spleen, cardiac, inflammation, neural-network

## Abstract

Stroke, a serious systemic inflammatory disease, features neurological deficits and cardiovascular dysfunction. Neuroinflammation is characterized by the activation of microglia after stroke, which disrupts the cardiovascular-related neural network and the blood–brain barrier. Neural networks activate the autonomic nervous system to regulate the cardiac and blood vessels. Increased permeability of the blood–brain barrier and the lymphatic pathways promote the transfer of the central immune components to the peripheral immune organs and the recruitment of specific immune cells or cytokines, produced by the peripheral immune system, and thus modulate microglia in the brain. In addition, the spleen will also be stimulated by central inflammation to further mobilize the peripheral immune system. Both NK cells and Treg cells will be generated to enter the central nervous system to suppress further inflammation, while activated monocytes infiltrate the myocardium and cause cardiovascular dysfunction. In this review, we will focus on microglia-mediated inflammation in neural networks that result in cardiovascular dysfunction. Furthermore, we will discuss neuroimmune regulation in the central–peripheral crosstalk, in which the spleen is a vital part. Hopefully, this will benefit in anchoring another therapeutic target for neuro-cardiovascular dysfunction.

## Highlights

- The cardiovascular-related neural network mediated by microglial inflammation contributes to cardiovascular dysfunction.
- The central and peripheral organs are connected *via* brain-derived antigen delivery and the spleen's immune response.
- Spleen-targeted neuroimmunomodulation may improve cardiovascular dysfunction after stroke.

## Introduction

Stroke is focal neurological dysfunction caused by a hemorrhage or infarction in the brain, retina, spinal cord, or subarachnoid space. Severe neurological dysfunctions, including unilateral weakness, numbness, loss of vision, diplopia, ataxia, and non-upright vertigo, are the common causes of death. Even worse, an injury caused by a secondary stroke is more serious with higher risks (Hankey, 2014). Approximately 25% of patients died within 1 year and only 50% survived for 5 years (Hankey, 2017).

Growing evidence suggests that stroke would not only lead to neurological dysfunction, but also cause systemic inflammatory response, immune dysregulations, and further damage to peripheral organs (Saand et al., 2019). In other words, damage may spread to the heart, lungs, gastrointestinal tract, liver, kidneys, spleen, and bone after stroke (McDonald et al., 2020). Although most patients with stroke die of neurological damage, cardiovascular complications appear to be the second major reason for death after a stroke (Chen et al., 2017). However, we still do not know whether cardiovascular diseases are unrelated or secondary to stroke. Diffusion tensor imaging (DTI) can be used to view the fibrous connection between the cardiovascular-related nucleus, which forms a neural network. The release of inflammatory molecules after stroke will destroy the neural network and cause serious neurological complications (Ghosh and Basu, 2012). Most importantly, activated microglia would engage in short-term (acute stage) and long-term (chronic stage) neuroinflammation after stroke (Wang et al., 2021). Our review will focus on how microglia contribute to the cardiovascular-related neural network.

In addition to the heart, the spleen is also regulated by neural networks and leads to the peripheral immune response of stroke. The spleen will release inflammatory cells to infiltrate cardiomyocytes and cause dysfunction. The removal of the spleen can alleviate neurodegeneration and other cellular degeneration of the body during an ischemic stroke (Pennypacker and Offner, 2015). Current therapeutics and interventions for stroke rehabilitation can be further improved to live up to patients' needs (Winstein et al., 2016). We need to elucidate central and peripheral interactions to investigate the possible pathway targets and explore effective strategies for stroke treatment.

A literature search was performed on PubMed and Web of Science databases without restrictions to publication types and languages between the years 2000 and 2021. The following terms are not limited to an individual or multiple combined queries: stroke, microglia, neuroinflammation, cytokine, neural network, cardiovascular, spleen, and crosstalk. We also studied the references listed in the literature found in accordance with the above search principles and selected the ones we thought were useful and relevant. We tried to demonstrate the possible mediators and complex interaction mechanisms between microglia polarization and heart injury, but our research has limitations as there were few relevant experimental or clinical studies.

## Cardiovascular dysfunction-related neural network after stroke

Neuro-cardiovascular dysfunction refers to cardiovascular disease caused by the disruption of the neural network after stroke, which generally includes blood pressure variability, arrhythmias, unstable angina, myocardial infarction, or heart failure. Although neurological defects are the leading cause of death after stroke, the number of patients who die of heart damage cannot be ignored. Recent studies have even proposed “stroke-heart syndrome” (Scheitz et al., 2021). More and more studies believe that stroke will cause not only neuroinflammation, but also systemic damage. Some specific detection biomarkers of heart injury will change significantly, and can even be used as a stratification of post-stroke risk (Chen et al., 2017). Scheitz et al. (2021) provide prognostic implications of elevated cardiovascular troponin levels in patients with acute ischemic stroke. Approximately 30–60% of patients have elevated CTn levels at admission, which is higher than expected in older adults without acute stroke. The CTn level of 15% of patients was increased even beyond the threshold used in the emergency department to classify patients with chest pain as “suspected myocardial infarction.” Suleiman et al. (2017) also reported that the mean values of serum cTnT and CK-MB (creatine kinase) were higher in patients with acute ischemic stroke compared to controls. Although there are no clear guidelines to clarify myocardial markers as post-stroke risks, cardiac functions in patients with stroke are supposed to be comprehensively evaluated.

Many brain regions that control emotion, stress, and homeostasis, including the anterior insula, anterior cingulate cortex, amygdala, hypothalamus, periurethral gray matter, parabrachial nucleus, and medulla, would influence the heart rate and cardiovascular contractility through the sympathetic and parasympathetic nervous systems (Palma and Benarroch, 2014). The adverse consequences of various neurological diseases on the heart emphasize the need for a better understanding of the functional, anatomical, and neurochemical mechanisms of cardiovascular nerve control, especially severe arrhythmias and cardiovascular injuries following neurotraumatic events (Palma and Benarroch, 2014). The sympathetic and parasympathetic nervous systems, characterized as powerful regulators of the cardiovascular system, remain largely unknown at the molecular and functional diversity levels of their constituent neurons and circuits (Palma and Benarroch, 2014).

**Figure 1:**
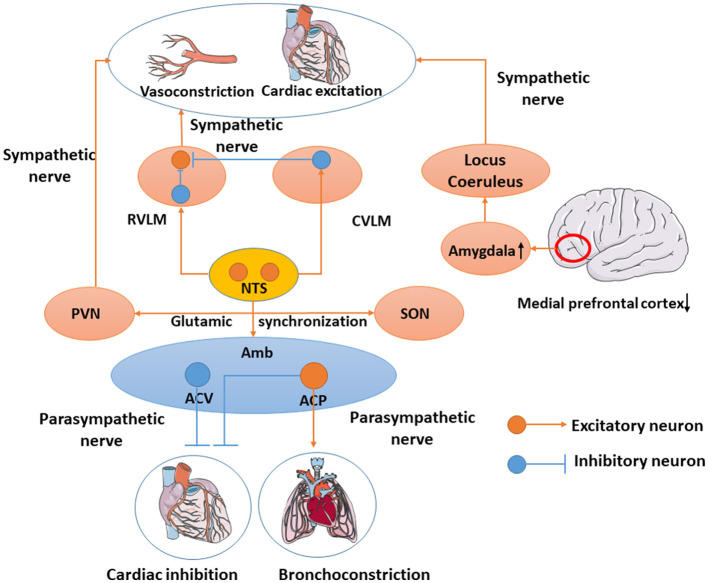
Cardiovascular-related neural network. Amb includes ACV and ACP. ACV will sense changes in peripheral blood pressure and regulate cardiovascular function, whereas ACP induces bronchoconstriction simultaneously. NTS will project an excitatory projection onto the Amb to activate the vagus nerve and inhibit cardiovascular activity. NTS also activates CVLM and RVLM. In the meantime, CVLM would inhibit RVLM to inhibit vasoconstriction and cardiovascular excitation. Overactivation of the amygdala leads to the continued activation of the noradrenaline-producing locus coeruleus and chronic activation of the sympathetic nervous system. PVN regulates the sympathetic nerve and SON synchronizes with PVN through the glutamic acid circuits.

Cardiovascular parasympathetic effluents originate from the brain stem preganglionic neurons, mainly located in the ambiguus (Amb), and a few cardiovascular parasympathetic nerves located in the motor nucleus of the dorsal vagus nerve. Avin Veerakumar's recent dissection of cardiovascular parasympathetic control circuits in mice revealed cardiovascular innervation neurons in the brainstem. Amb is composed of two molecules that are anatomically and functionally distinct subtypes called ambiguus cardiopulmonary (ACP) and ambiguus cardiovascular (ACV). ACV will sense changes in the peripheral blood pressure and selectively activate the cholinergic cardiovascular ganglion neurons, which regulate the cardiovascular function, whereas ACP is recently found and activates the dive reflex which can also induce bronchoconstriction simultaneously (Veerakumar et al., 2022). Ching-Yi Tsai used DTI to revise the baroreflex neural circuits that consist of nucleus tractus solitarii (NTS) and Amb or the rostral ventrolateral medulla (RVLM) and the caudal ventrolateral medulla (CVLM). The NTS will project an excitatory projection onto the Amb to activate the vagus nerve and inhibit cardiovascular activity. In addition, NTS also regulates blood pressure by activating the CVLM and RVLM. Simultaneously, the CVLM also projects onto the inhibitory projective fibers to RVLM (Tsai et al., 2019) to restrain vasoconstriction and cardiovascular excitation. Stressful conditions in the brain can reduce activity in the medial prefrontal cortex, leading to the overactivation of the amygdala. This leads to the continued activation of the noradrenaline-producing locus coeruleus and chronic activation of the sympathetic nervous system both of which promote aseptic inflammation (Yang et al., 2021). Glutamic acid circuits in the hypothalamus link the contralateral supraoptic nucleus (SON) and the ipsilateral paraventricular nucleus of the hypothalamus (PVN) large cell neurons together and activate their synchronization. The PVN and RVLM are key components of the neural network that generate and regulate sympathetic nerve activity (Boudaba and Tasker, 2006) (Figure 1).

## Microglia-modulated neural networks in inflammatory mechanisms

Previous studies have suggested that microglia affect the peripheral organs by regulating the autonomic nerve, especially through sympathetic and parasympathetic regulation (Li Y. et al., 2020), i.e., the hypothalamic-pituitary-adrenal (HPA) axis and catecholamine surge (Chen et al., 2017). Furthermore, modulation of microglial activity has been shown to regulate the incidence and progression of cardiovascular diseases. Microglia have been indicated as a potential regulator of hypertension, myocardial infarction, and ischemia/reperfusion injury (Wang et al., 2022). The dysregulation of cytokine secretion and neurotoxic metabolites released from microglia seriously damage neural-network activity, leading to pathological outcomes (Li Y. et al., 2020). M1 microglia are detected immediately within hours of stroke, stimulating the pro-inflammatory mechanisms to mediate the neurotoxic effects, while M2 microglia occur a few days after stroke, mediating the anti-inflammatory mechanisms to exert neuroprotective effects (Wang et al., 2021). Therefore, future exploration of the molecular linkage of microglia may provide various therapeutic targets.

Toll-like receptors belong to the innate immune receptor family, which play a significant part in neuroinflammation (Fang et al., 2013). The activation of TLR4 in microglia could initiate the myeloid differentiation primary response protein 88 (MyD88) pathway. The Toll-receptor-associated activator of the interferon (TRIF) pathway is triggered to express the nuclear factor-κB (NF-κB) and release inflammatory cytokines such as TNF-α and IL-6 (Wang et al., 2021). AngII Type 1 receptor (AT1R), widely expressed by various central nervous system cell types, directly affects neuronal activity by binding neuron AT1R or affecting the endothelial cells and perivascular macrophages. However, endogenous AngII will promote the interactions between AT1R and TLR4, thereby activating microglia and disrupting the BBB. Tac-242 (resatorvid), a specific TLR4 inhibitor, will improve neurogenic hypertension (Mowry et al., 2021). Masson et al. (2015a) also found the expression of TLR4 in PVN and that acute LPS treatment would influence heart rate. Therefore, therapy targeting TLR4 may be a potential strategy for alleviating neurogenic cardiovascular disease.

The high-mobility group protein box 1 (HMGB1) and the advanced glycation end-product receptor (RAGE) are involved in microglia polarization and hypertension (Li et al., 2022). The ubiquitous protein in nucleoprotein is known as the high-mobility group protein box 1 (HMGB1). This nuclear protein is released into the peripheral environment to promote inflammation (Andersson et al., 2018) after cell activation, stress, injury, or death. Upon neuroinflammation, the microglia secrete HMGB1 that can activate TLR4 expression and then promote the secretion of inflammatory cytokines through the NF-κB pathway, thus regulating the polarization of microglia (Li et al., 2022). In addition, studies have shown that HMGB1 can increase TNF-α and IL-1β to aggravate neuroinflammation (Yang et al., 2017). In spontaneously hypertensive rats, HMGB1, IL-6, and TNF-α levels were higher in the PVN (Masson et al., 2015b), which suggest the significant role of microglia involved in the cardiovascular network.

P2X receptor, a subtype of P2 receptor, located on the cell membrane of microglia is an ion-gated channel that is activated by the ATP in microglia. Some studies have shown that mice with knocked-out P2X receptor genes express less TNF-α and IL-1β and reduce neuroinflammation (Subauste, 2019). The P2X7 receptor is the key to microglia-mediated cardiovascular injury. In an animal experiment, the P2X7 receptor was co-located with the microglia in the PVN of mice and the increased ATP content in the PVN leads to an increase in P2X7R expression. Activated microglia release TNF-α and IL-1β that stimulate the release of oxytocin and vasopressin through the P2X7 receptor. Oxytocin and vasopressin participate in the regulation of the cardiovascular system by activating the sympathetic nerve (Du et al., 2015).

**Figure 2:**
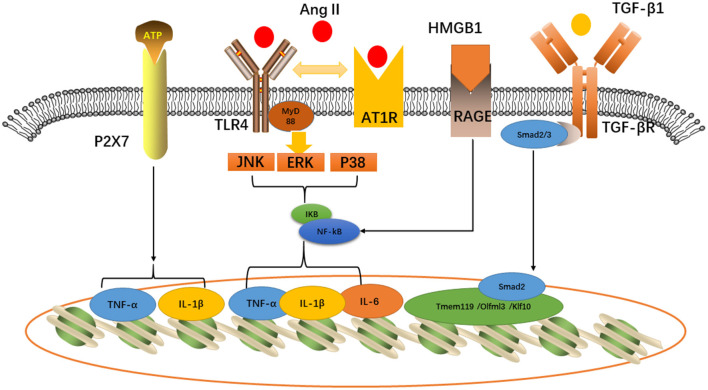
Inflammatory pathways related to cardiovascular diseases expressed in microglia: P2X7, TLR4-AT1R, and HMGB1-RAGE would activate TNF-α, IL-6, and IL-1β, while TGF-β would activate Tmem119/Olfml3/Klf10 induce microglia maturation.

TGF-β1 stimulates the M2 phenotype microglial transition that contributes to play an anti-inflammatory function. Tissue regeneration and neurological recovery are promoted by the TGF-β/Smad2/3 pathway in mice that have gone through stroke (Zhang et al., 2021). Furthermore, TGF-β can suppress hypertension by modulating microglia (Li Y. et al., 2020). In normotensive rats, TGF-β would inhibit neuroinflammation, the kidney norepinephrine level, and blood pressure, which are mediated through microglia (Li et al., 2017). Tmem119, Olfml3, and Klf10 can be direct Tgfβ1-Smad2 target genes, which are associated with microglia maturation and maintenance (Spittau et al., 2013; Attaai et al., 2018; Neidert et al., 2018). Therefore, TGF-β is a unique microglial signature that provides the possibility for targeting microglia for treatment (Butovsky et al., 2014) (Figure 2).

## Neuroimmune regulation in central-peripheral crosstalk

### Brain-derived antigen through the brain–blood barrier and the lymphatic pathway

Endothelial cells, astrocyte end-feet, and pericytes compose the BBB (Sweeney et al., 2019) that could prevent neurotoxic plasma components, blood cells, and pathogens from entering the brain and modulate the molecules into and out of the central nervous system (CNS) (Sweeney et al., 2019; Zhang et al., 2020). Increased BBB permeability is a major pathophysiological change after stroke that promotes the entry of inflammatory molecules. Interestingly, the BBB permeability is increased not only during inflammatory responses. Hendy and Hall (2019) reported in their study that up to 50% of patients with cardiovascular disease showed a temporary increase in BBB permeability after surgery and other studies also show that peripheral leukocytes (Greenwood et al., 2011), cytokines, chemokines, proteases, and adhesive proteins (Rahman and Dandekar, 2021) may change when the BBB gets disrupted which suggests that the brain–heart interaction is likely to play a role in immune molecules passing through the BBB. In addition, cardiovascular diseases are likely to be one of the systemic inflammatory reactions of stroke.

**Figure 3:**
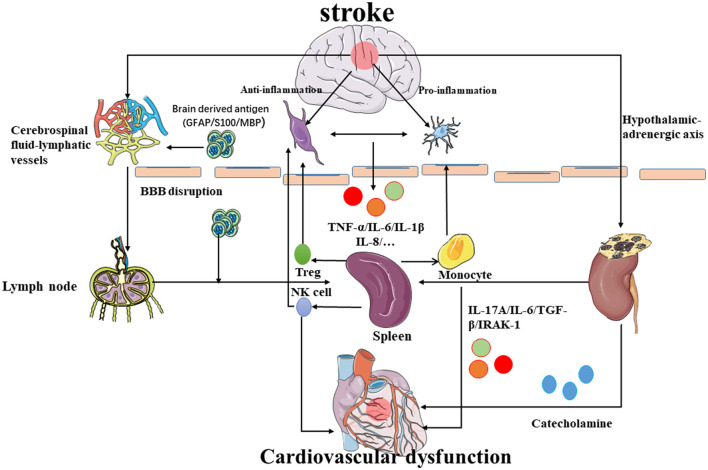
Possible mechanism of brain–spleen–heart crosstalk: after stroke, the permeability of the blood–brain barrier increases, and the immune cells and inflammatory molecules in the brain reach the peripheral lymph nodes and spleen through the BBB to further regulate the occurrence of immune inflammation. The inflammatory molecules released by spleen activation mediate myocardial damage. Simultaneously, the spleen also releases NK cells and Treg cells into the brain to inhibit neuroinflammation while the monocytes would promote neuroinflammation. In addition, the inflammatory reaction near the hypothalamus can also promote the hypothalamic adrenaline axis to regulate the immune response of the spleen and heart damage.

Injured astrocytes, neurons, and oligodendrocytes (Zhang et al., 2020) release brain-derived antigens such as glial fibrillary acidic protein, S100, and myelin basic protein (MBP) (Chen et al., 2017). Hochmeister et al. (2008) injected antigens into both the CNS and cerebrospinal fluid and found them in the deep cervical lymph nodes, mesenteric lymph nodes, and spleen. Furthermore, antigen-presenting cells (APCs) and soluble anti-inflammatory proteins can also be detected in the peripheral lymph nodes during inflammation (Planas et al., 2012). Although the brain has no lymphatic vessels, the brain antigens and lymphocytes break through the BBB and flow out along the arterial wall to the cervical lymph nodes (Clapham et al., 2010). However, there is also communication between the cerebrospinal fluid and lymphatic vessels, and other antigenic substances can also enter the peripheral lymph nodes in this way (Johnston et al., 2004). Latest research shows that the high-resolution real-time imaging technology makes the landmark discovery of the peridural lymphatic system of the brain possible, which provides a deeper understanding dimension for neuroimmunology, and the central nervous system will no longer be an immune-privileged area (Negi and Das, 2018) (Figure 3).

### Spleen: Peripheral immune response in brain–body interaction

A specific brain network exists that connects to the spleen through the splenic nerve and produces a rhythmic discharge; even severing the link between the spleen and the central nervous system may treat refractory hypertension (Carnevale et al., 2016), which indicates the emerging role of neural networks in systematic inflammation. A neural network modulates the production of T cells that in turn produces the neurotransmitters to inhibit innate immunity (Rosas-Ballina et al., 2011). Although it has been proposed that the brain–spleen connection may be an important mechanism of neuroimmunity in cardiovascular injury, more experiments need to be conducted to prove it.

Splenic activation is associated with neuroimmune activation to respond to systemic inflammation and regulate the immune system and impaired neurological recovery (Saand et al., 2019). The spleen has been proposed to play an important role in the brain–heart crosstalk (Chen et al., 2017). Most studies have preliminarily shown that the spleen is involved in brain injury after ischemic stroke and hemorrhagic stroke (Ajmo et al., 2008). A reduction in splenic volume from 48 to 96 h after a stroke has been observed (Saand et al., 2019) in an MCAO model. Interestingly, the splenic volume will return to its previous level around the 10th day after stroke. Experiments in animals have shown that administering simvastatin immediately after the stroke can reduce spleen atrophy and splenocyte apoptosis in a mouse MCAO model, thereby inhibiting the expression of brain IFN-γ and reducing the risk of pulmonary bacterial infection (Lee et al., 2008) and proving that there is a close relationship between spleen and stroke. As the main peripheral immune organ of the human body, the spleen may be connected with the brain through the immune system, such as various immune cells and inflammatory molecules (Saand et al., 2019). Central immune cells have been shown to escape from the central to cervical lymph nodes through the cerebrospinal fluid lymphatic pathway and it is worth noting that many central immune cells have also been found in the spleen (Hochmeister et al., 2008).

Some studies conducted in rats have shown that the cells of the spleen have undergone great changes after stroke while the number of neutrophils and macrophages in the spleen did not change significantly after stroke. The T cells and B cell levels of the spleen decrease significantly after stroke, but the false spleen did not undergo changes in the control group. Liu, Q found that spleen macrophages, T cells, and B cells were negatively correlated with the neurological dysfunction score (NDS), suggesting that these spleen immune cells may contribute to stroke recovery. Some studies believe that spleen-derived monocytes are consistent with the increase in monocytes in the brain after stroke and play an anti-inflammatory role in ischemic stroke (Kim et al., 2014; Han et al., 2020).

NK cells were used as a bridge in an animal experiment using MCAO mice that can be shown that the activation of catecholamine and HPA axis may be the reason for the spatiotemporal fluctuation of the number of NK cells in the central nervous system and peripheral system. After a stroke, the activation of catecholamine and the HPA axis can reduce the number of peripheral NK cells through the JAK-STAT signal pathway and induce the NK cells of the peripheral system to enter the brain through the blood–brain barrier (Liu et al., 2017). In another period, through the regulation of the brain immune axis, spleen atrophy and the spleen-mediated anti-inflammatory mechanism began to protect nerve cells by increasing the Treg cells (Offner et al., 2006a,b; Vendrame et al., 2006). Treg cells are released from the spleen, which can regulate the immune system and inhibit the inflammatory response and autoimmunity. Simultaneously, the presence of Tregs can induce the polarization of microglia to the M2 type, which is more conducive to reducing the damage caused by the inflammatory response (Zhou et al., 2017). The expansion of the number of Tregs in the brain after ischemic stroke is considered to be one of the endogenous anti-inflammatory mechanisms (Yang et al., 2014) and its function has been confirmed (Offner et al., 2006b; Urra et al., 2009). However, the effect and mechanism of Tregs in ischemic stroke still need more experimental research. Interestingly, in splenectomized animals, the neuroprotective effects seem to disappear which suggests that the spleen is essential for the mechanisms of these neuroprotective strategies. Studies have shown that spleen-derived Tregs cells can also prevent the activation of the TLR4 and NF-κB pathways in the brain to reduce the inflammatory response of the central system (Kim et al., 2015; Wang et al., 2016). In general, regulation of the brain immune axis would activate the spleen-mediated peripheral immune response, which can be fed back to the brain to achieve the effect of inflammation or anti-inflammation (Venkat et al., 2018).

The spleen correlated with the inflammation closely, and hemodynamic congestion, and sympathetic stimulation would affect the size of the spleen (Maeda et al., 2021). However, the inflammation mechanisms require more investigation. Dunford et al. (2017) used fluoro-18-fluorodeoxyglucose (18F-FDG) PET/CT to investigate the spleen–heart axis. Monocyte activation is the key to heart damage, and the processing of antigens by the spleen enhances their activation and promotes the immune-mediated injury response (Ismahil et al., 2014). Some studies have shown that IL-17A, which may be derived from pathogenic Th17 cells and effector/memory T cells, in the spleen and blood would prominently affect the heart (Zamora et al., 2021). Similarly, the release of inflammatory factors into the blood after heart injury will also lead to the remodeling of spleen tissue (Jahng et al., 2016). A mice model study concluded that the release of proinflammatory cytokines and inflammatory cells infiltrate the myocardium after ICH leading to cardiovascular pathological remodeling and cardiovascular dysfunction (Zhao et al., 2019; Yan et al., 2020). As the regulator of peripheral immunity, the spleen will continue to release inflammatory cells with the activation of the peripheral immune response which will infiltrate the myocardium and result in acute or chronic cardiovascular dysfunction (Zhao et al., 2019). After splenectomy, the number of cardiovascular-infiltrated macrophages and leukocytes decreased significantly and the ICH-induced cardiovascular dysfunction was improved (Mebius and Kraal, 2005; Li W. et al., 2020). In animal models, IL-6 inhibition can significantly reduce cardiovascular insufficiency, inflammation, and fibrosis (González et al., 2015). The ICH and splenectomy groups significantly reduce MCP-1 expression and macrophage and monocyte infiltration compared with the ICH group without treatment. Therefore, spleen-mediated immune responses may be the main cause of cardiovascular dysfunction after ICH, which may be a bridge of the brain–heart interaction after stroke (Li W. et al., 2020).

### Promising treatments for cardiovascular dysfunction after stroke

Neuroimmune regulation has long been neglected by cardiologists. A pioneering work in David Harrison's lab (Harrison, 2013) has brilliantly demonstrated that lymphocytes are needed to raise blood pressure and also Tracey (2009) found that stimulating the nerve reflex in the spleen protected the mice from septic shock. Specific regions of the perception of peripheral circulation can integrate the immune and vascular lesion reactions and discover the key role of the spleen. This secondary lymphoid organ produces prime immune cells that are subsequently recruited to target organs and are crucial for blood pressure regulation (Carnevale and Lembo, 2018). However, this discovery still needs further investigations and it is necessary to map the cerebral splenic nerve and the cerebral cardiosympathetic nerve with microneurography.

ANS activation, brain-derived antigen, and activation of chemokines have been proven to play crucial roles in brain–spleen after stroke (Wang et al., 2019). Since the spleen is the neuroimmunomodulatory center of the systemic inflammatory response after stroke, its treatment strategy has received increasing attention. While splenectomy can help to reduce the infarct size after stroke (Saand et al., 2019), Carvedilol, a beta blocker, can significantly reduce infarct size due to the massive release of catecholamines on the splenic nerve after stroke that activates the immune function of the spleen (Ajmo et al., 2009). Simvastatin can also ameliorate peripheral immunodepression after stroke by attenuating spleen atrophy (Jin et al., 2013). However, immunomodulatory regulation is an important means of activating the spleen. Stem cell therapy targeting the spleen is a promising strategy for treating systemic inflammatory responses after stroke. Intravenously injected stem cells modulate the inflammatory response by targeting the brain and spleen after stroke, and microglia also transform from the M1 proinflammatory phenotype to the M2 anti-inflammatory phenotype, providing a favorable environment for nerve regeneration and angiogenesis (Wang et al., 2019). In addition, we found that selective thermal ablation of splenic nerves prevents T-cell expulsion (Carnevale et al., 2016). Interleukin (IL)-33 has been found to increase IL-4, IL-10, and TGF-beta levels in the spleen which would exert the M2-phenotype microglia and promote nerve regeneration (Xiao et al., 2019) (Table 1).

**Table 1:** Neuroimmune regulation targeting spleen.

**Theraputic strategy**	**References**
Splenectomy	Saand et al., 2019
Carvedilol	Ajmo et al., 2009
Simvastatin	Jin et al., 2013
Stem cell therapy	Wang et al., 2019
Ablation of splenic nerves	Carnevale et al., 2016
Interleukin (IL)-33	Xiao et al., 2019

## Conclusion

**Figure 4:**
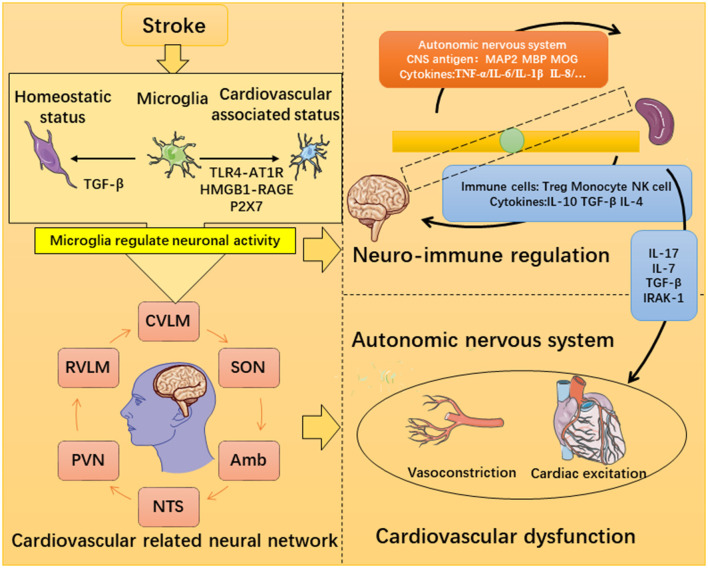
Possible mechanism of microglia-mediated inflammatory destruction of neuro-cardiovascular dysfunction.

This is a review of the mechanisms of severe neuroinflammation and cardiovascular dysfunction after stroke (Figure 4). Degeneration products generated after stroke will stimulate specific immune receptors of microglia and also lead to a disruption of the neural network and cause systemic inflammation. Cardiovascular dysfunction is the most serious injury in peripheral organs, and the microglia-mediated inflammatory response can regulate ANS activation to impact the peripheral organs. More importantly, brain-derived antigens and other cytokines can reach the spleen through the BBB and the lymphatic pathway, thus influencing the immune response of the spleen. Monocyte cells can infiltrate the myocardia to mediate heart damage. Treg cells and NK cells can also enter the central nervous system to inhibit the occurrence of central nervous inflammation, while the monocytes have the opposite effect. Therefore, the crosstalk between the brain, spleen, and heart may be an important mechanism of cardiovascular dysfunction after stroke, and the spleen plays an important role in it. The treatment of neuroimmunomodulation targeting the spleen has been investigated but effective strategies for regulating cardiovascular-related neural networks still need development.

## Author contributions

JW and HH initiated this project. JD, CC, and SX performed the literature research and contributed to the original draft. DS and WP participated in the revision of the manuscript. All authors contributed to the article and approved the submitted version.
